# Experimental insights into elasto-inertial transitions in Taylor–Couette flows

**DOI:** 10.1098/rsta.2022.0131

**Published:** 2023-03-20

**Authors:** T. Boulafentis, T. Lacassagne, N. Cagney, S. Balabani

**Affiliations:** ^1^ Department of Mechanical Engineering, FLUME, University College London (UCL), London WC1E 7JE, UK; ^2^ IMT Nord Europe, Institut Mines-Télécom, Univ. Lille, Centre for Energy and Environment, Lille F-59000, France; ^3^ School of Engineering and Materials Science, Queen Mary University of London, London E1 4NS, UK

**Keywords:** Taylor–Couette flow, elasto-inertia, experiments

## Abstract

Since the seminal work of Taylor in 1923, Taylor–Couette (TC) flow has served as a paradigm to study hydrodynamic instabilities and bifurcation phenomena. Transitions of Newtonian TC flows to inertial turbulence have been extensively studied and are well understood, while in the past few years, there has been an increasing interest in TC flows of complex, viscoelastic fluids. The transitions to elastic turbulence (ET) or elasto-inertial turbulence (EIT) have revealed fascinating dynamics and flow states; depending on the rheological properties of the fluids, a broad spectrum of transitions has been reported, including rotating standing waves, flame patterns (FP), and diwhirls (DW). The nature of these transitions and the relationship between ET and EIT are not fully understood. In this review, we discuss experimental efforts on TC flows of viscoelastic fluids. We outline the experimental methods employed and the non-dimensional parameters of interest, followed by an overview of inertia, elasticity and elasto-inertia-driven transitions to turbulence and their modulation through shear thinning or particle suspensions. The published experimental data are collated, and a map of flow transitions to EIT as a function of the key fluid parameters is provided, alongside perspectives for the future work.

This article is part of the theme issue 'Taylor–Couette and related flows on the centennial of Taylor’s seminal *Philosophical Transactions* paper (part 1)'.

## 1. Introduction

Taylor–Couette (TC) flow, the flow between two concentric cylinders, has received considerable fundamental and practical interest over the last 100 years. It has served as a common rheological flow and a paradigm to study hydrodynamic instabilities since the work of [1]—due to its apparent geometrical simplicity—and enjoys numerous applications in manufacturing and process engineering: bioreactors for cell cultivation and tissue engineering [2–4], nanosensor fabrication [5], water purification [6], graphite exfoliation into graphene [7], starch hydrolysis [8,9], shearing of proteins [10,11] to name but a few.

**Figure 1.**
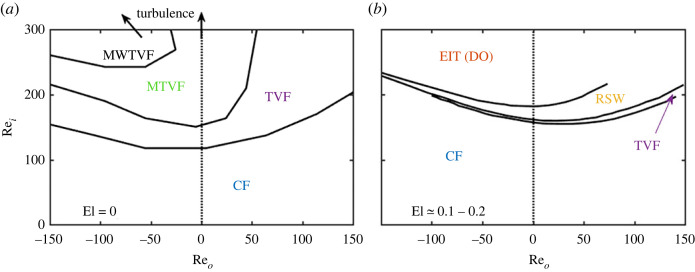
A map of flow states for co-rotating and counter-rotating TC flow in the Newtonian case ((a) derived from [14], elastic number El=0) and in a viscoelastic case ((b) derived from [15], elastic number El≃0.1–0.2) for inner and outer cylinder Reynolds number, Re${\;}_{i}\in [0:300]$ and Re${\;}_{o}\in [-150:150]$, respectively. Those illustrate the two very different populations of expected flow states. Acronyms for flow states are defined later in the text. (Online version in colour.)

Although the stability of the TC flow has been exploited to measure shear viscosity [12], Taylor [1] was the first to identify a primary bifurcation from the laminar Couette flow of a Newtonian fluid into a new state characterized by the appearance of toroidal vortices, later named after him: Taylor–Vortex flow (TVF). Following his seminal work, several higher-order instabilities have been identified for Newtonian fluids sheared in the gap of co- and counter-rotating cylinders [13] as the rotational speed is varied; these are summarized in figure 1a, reproduced from [14]. A wide variety of flow states is evident for different ratios of rotational speeds for the inner and outer cylinders $\Omega_{i}/\Omega_{o}$ and for increasing rotational speeds, eventually leading to inertial turbulence, a topic of immense interest in the fluids community over the years [16,17].

In many of the aforementioned applications of the TC flow, the fluids are non-Newtonian in nature, i.e. they exhibit a nonlinear relationship between stress and strain rate [18]. Nonlinear interactions can have a profound effect on the range of fluid instabilities encountered [19] (see figure 1). One of the most striking manifestations of this is the turbulent drag reduction phenomenon (TDR) in pipe flows, whereby the addition of small amounts of polymer alters the nature of turbulence and reduces frictional drag [20,21]. Non-Newtonian, polymer solutions are known to induce elastic instabilities due to the non-zero normal stress differences $N_{1}$ and $N_{2}$ (termed first and second normal stress differences, respectively). These generate a force in the direction of the streamline curvature for curvilinear flows, leading to well-known elastic phenomena such as the Weissenberg effect (rod climbing) [22]. Such phenomena combined with the richness of TC flow and the complexity and structural properties of viscoelastic fluids give rise to a wealth of new flow states and instabilities leading to elastic or elasto-inertial turbulence (EIT) [23,24].

In contrast to ET, which appears only in curvilinear flows due to hoop stresses (excluding the numerical works of [25]), EIT also appears in unperturbed straight flows (e.g. pipe/channel and jet flows) [26–29]. The onset and structure of EIT in pipes/straight channels have been studied extensively, both experimentally and numerically, since its first observation by [27] revealing coherent structures like near-wall extensional streaks/chevron-type patterns [26,30] or arrowhead regimes [31,32]. The motivation for some of these studies is the fact that adding polymers to a pipe flow has been long known to reduce drag [33]; this is a polymer concentration-dependent phenomenon reaching a universal asymptote so-called maximum drag reduction (MDR). It was not until later that polymer drag reduction and MDR were attributed to the onset of EIT [20,21,26,27,30,34]. The latter coincides with a weakening of vortices aligned in the streamwise direction (which are effective at transmitting momentum between the freestream and the boundary layer) and a growing dominance of vortices aligned perpendicular to the flow [21].

In TC flows, ET and EIT have not been fully characterized experimentally to allow qualitative and quantitative comparisons of mechanisms and structures with pipe/channel flows. Samanta *et al.* [27] found that the onset of EIT in the pipe flow was independent of the perturbation applied, suggesting that it was associated with a supercritical transition (supported by later numerical and experimental works [35,36], while [15] found that the transition to EIT in TC was hysteretic, i.e. a subcritical transition). This might imply significant differences between the dynamics of EIT in pipe and TC flows, and the link between them remains unclear.

Choueiri *et al.* [26] argued that the common property of EIT in all flow systems is the longer spatial scales and the more coherent, uniformly distributed structures. Several researchers have examined the turbulent decay exponent, k, in EIT in channel and TC flow. In the channel flow, the estimates for k include −14/3 or −11/3 [37], −3 [38], and in TC, values of −14/3 [39], −3.5 [40] and −3 [41] have been reported. The estimates of k found in various studies are quite scattered but fall in the range −14/3 to −3 for both systems. Therefore, it is not possible to draw any conclusion as to the universality of EIT from these studies, but further work may yield more insight into this question.

Elastic instabilities and turbulence in TC flow have been the subject of research for several decades; a comprehensive review can be found in [19]. The interaction of elasticity-driven effects and inertia on the other hand is less understood and has recently gained significant attention, both numerically and experimentally. In this review, we discuss recent experimental attempts to elucidate the effects of elasticity and inertia on the flow transitions and their modulation by rheological complexity. The review is structured as follows: §2 presents the key parameters controlling viscoelastic TC flows and transitions, the non-dimensional numbers typically used to describe/generalize the results and the experimental methods commonly employed to characterize these flows. Section 3 presents an overview of the role of elasticity on the transitions to turbulence; the cases of zero-elasticity and purely elastic transitions are considered first to provide a measure of comparison, followed by the combined effect of inertia and elasticity. The effects of more complex rheologies, such as shear-thinning and particle suspensions, on EIT transitions are described in §4. The published experimental works are summarized in §5; a flow transition map is provided and future directions are discussed.

## 2. Flow configuration, key parameters and experimental methods

### (i) Flow geometry and non-dimensional parameters

**Figure 2.**
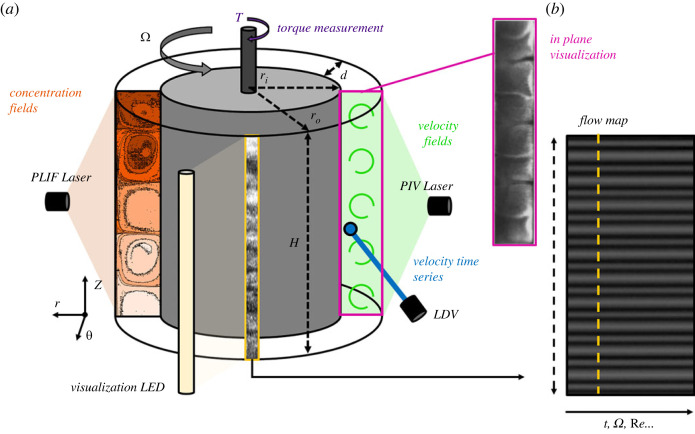
Schematic representation of common experimental techniques used to investigate TC flows. (a,b) Planar laser-induced fluorescence (PLIF) is used to assess mixing performances (e.g. [42,43]); flow vislualization allows us to easily probe the flow structure and construct spatio-temporal diagrams (e.g. [15,23,44,45]); torque measurement can detect the onset of secondary flows and evaluate friction properties of flow states [46–48]; particle image velocimetry (PIV) is designed to measure flow velocity and accurately describe flow features within the gap (e.g. [49]); alternatively, qualitative in-plane visualization can be performed using rheoscopic flakes similar to the ones employed for the spatio-temporal analysis [50–52]; laser Doppler velocimetry (LDV) is used to measure fluid velocity locally with a high temporal resolution [53]. (Online version in colour.)

A typical TC geometry is shown in figure 2. It is fully characterized by the inner and outer cylinder radii, $r_{i}$ and $r_{o}$, respectively, and the height H (see figure 2). These are typically presented in various non-dimensional forms, using the radius ratio, $\eta_{\text{cell}}=r_{i}/r_{o}$, the aspect ratio, Γ=H/d (where $d=r_{o}-r_{i}$ is the gap between the cylinders) and the curvature ratio $\epsilon =d/r_{i}$. Based on this set of parameters, the TC setups can be separated into high aspect ratio (Γ>20) and low aspect ratio (Γ<20) [48]. TC cells with $\eta_{\text{cell}}<0.5-0.65$ are generally considered wide/large gap and cells with $\eta_{\text{cell}}>0.7$ are considered narrow/small gap [48,54–57].

The most studied TC configuration involves a stationary outer cylinder and a rotating inner one. In this case, the flow can be described using a single non-dimensional number, the Reynolds number:

2.1 Re=ρΩiridη,

where ρ and η are the fluid density and viscosity, respectively. This is equivalent to the product of the viscous time scale, $t_{v}=\rho d^{2}/\eta$ and the nominal strain rate $\dot{\gamma }=\Omega_{i}r_{i}/d$. When the outer cylinder is also rotating in co- or counter-rotation, a Reynolds number based on its rotation speed $\Omega_{o}$ may be defined in a similar fashion [14,15]. An alternative form is commonly used, the Taylor number:

2.2 Ta=ρΩirid3η=Redri,

which incorporates the effects of curvature.

### (ii) Fluid parameters

In the experimental literature, complex, non-Newtonian fluids are typically generated through the addition of macromolecules to a Newtonian solvent. Different macromolecules have been used in the literature to generate viscoelastic solutions, including micellar solutions (e.g. [58–61]), T4 DNA [62] and polymers ([63] among many others), with the polymers being the most commonly employed.

A great variety of rheological properties can be achieved with polymer mixtures; the viscoelasticity of such mixtures depends strongly on the structure of the polymer chains and the solvent. The shear-thinning behaviour, typically observed, increases with polymer concentration and entanglement. However, it is also possible to retain the elastic properties of the fluid, while eliminating the shear-thinning behaviour, by using high-molecular-weight polymers in dilute solutions. These solutions are called Boger fluids [64,65].

**Figure 3.**
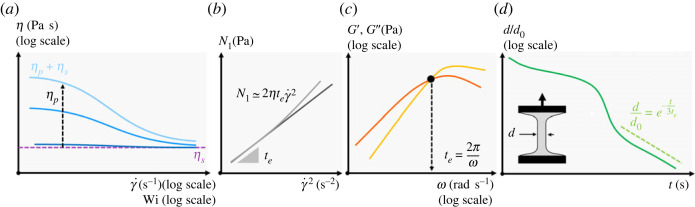
Illustration of key rheological characterization for the study of elasto-inertial TC flow. (*a*) Steady-shear characterization of potentially shear-rate dependent dynamic viscosity, from which an estimate of the elastic time scale can be occasionally derived (see the works of [45,66]). (*b*) Steady-shear characterization of the first normal stress difference as a way to estimate $t_{e}$ [52,67]. (*c*) Small amplitude oscillatory shear testing where the elastic time scale is estimated from the loss ($G^{\prime \prime }$) and elastic ($G^{\prime }$) moduli curves as in [23,44,68,69]. (*d*) Filament thinning tests typically performed on CaBER rheometers relating the elastic time scale to the dynamics of an elongated filament, as used in [15,47] among others. (Online version in colour.)

The effects of entanglement and shear-thinning are determined by the viscosity ratio:

2.3 $$\beta =\frac{\eta_{p}}{\eta_{s}},$$

where $\eta_{p}$ is the polymer contribution to the total solution viscosity and $\eta_{s}$ is the solvent viscosity. Boger fluids can thus be achieved when $\beta \overset{}{\to }0$ (see figure 3). However, this also depends strongly on the structure of the polymer chains. For example, polyethyleneoxide (PEO) and xanthan gum (XG) are semi-rigid and are known to produce strongly shear-thinning solutions [23,45,47,66,70], whereas polyacrylamide (PAAM) is often used to produce Boger fluids [67,69].

The elastic time scale, or relaxation time, of the viscoelastic fluids $t_{e}$ is typically assumed to be unique (the highest polymer relaxation time being the most relevant, as stated by [71]), shear-rate independent and is typically measured by small amplitude oscillatory shear rheology [23,44], measurement of first normal stress difference [46], extensional rheology [47], or estimated from viscosity steady-shear values [45,66] (see figure 3). It should be noted that methods based on shear rheology may be prone to discrepancy, as during shear flow, the polymers undergo a combination of elongational and rotational movement. This leads to a lower mean extension of the polymer chains as they both stretch and tumble [72] and thus, to an overestimation of the relaxation time compared to extensional methods [73]. Pereira *et al.* [74] used capillary breakup extensional rheometry (CaBER) to examine aqueous solutions of the three most common polymers used in experimental TC studies (PAAM, PEO and XG), for concentrations in the range of $10^{3}\text{--}10^{4}\;\mathrm{ppm}$. Unlike PAAM and PEO, XG exhibited very low, negligible, values of λ for all studied concentrations, attributed to the rigidity of its chains.

The effect of viscoelasticity is expressed via the Weissenberg number, Wi, which is defined as the ratio of elastic to viscous forces and for TC flow is given by:

2.4 $$\mathrm{Wi}=t_{e}\dot{\gamma }=\frac{t_{e}\Omega_{i}r_{i}}{d},$$

where $t_{e}$ is the relaxation time (elastic time scale) and $\dot{\gamma }$ is the nominal shear rate in the gap (TC gap or rheometer gap).

The ratio of the Weissenberg number (equation 2.4) to the Reynolds number (equation 2.1) is called elasticity number El:

2.5 El=WiRe=teηρd2

and is a measure of the relative importance of viscoelasticity. It must be noted that El depends only on the viscoelastic properties of the fluid and the geometry; it is not dependent on flow parameters like Wi. In a typical experimental elasto-inertial TC flow study, a combination of two among the three numbers, El, Re and Wi, are typically needed to characterize the flow.

### (iii) Flow characterization

A series of optical, non-intrusive methods have been mostly used to characterze viscoelastic TC flows. The most common one is flow visualization (see figure 2), which has proven to be a powerful tool to identify flow transitions and states. In this method, Kalliroscope flakes, such as mica flakes, are dispersed into the flow. Because of their highly anisotropic shape, mica flakes reflect light according to their orientation, indicating the direction of the flow [68,75]: high reflection implies strong azimuthal motion, whereas low-light intensity implies strong radial flow. The concentration has to be small enough (typically <0.1%) to have a minimal effect on the flow [76] and the fluid viscosity. The reflected light intensity, which highlights the flow structure, is then recorded using a camera. Some authors have opted for images of the whole test section to illustrate the flow patterns (e.g. [47]), whereas others record a small strip along the height of the TC cell (e.g. [15,44,69]). In the latter, the strip is averaged in the azimuthal direction, and all frames are compiled producing spatio-temporal maps that illustrate the evolution of the flow with time or any time-related control parameter (see figure 2b). Three different types of visualization experiments can be performed: (i) slowly accelerating the inner cylinder (ramp-up), (ii) slowly decelerating the inner cylinder (ramp-down), and (iii) keeping a constant rotational speed (steady-state). The acceleration/deceleration rate of the inner cylinder can greatly affect the dynamics of the system both qualitatively (different transitions) and quantitatively (variation of the critical Re at which they appear) as shown in [23,68,77]. In these works, to ensure quasi-static acceleration/deceleration of the fluids, the non-dimensional acceleration is used:

2.6 $$\Gamma_{0}=\frac{\mathrm{dRe}}{d\tau }=\frac{\rho^{2}r_{i}d^{3}}{\eta^{2}}\frac{d\Omega }{dt},$$

where $\tau =t/t_{v}$ is the non-dimensional time based on the viscous time scale $t_{v}=(\rho d^{2})/\eta$. For low values of $\Gamma_{0}<1$, the flow can be considered quasi-static and slower ramp-ups/downs would not yield any difference in the transitions. Alternative ramp protocols, such as a step-increase/decrease of the rotational speed [52], have been employed in published studies. Dutcher and Muller [15] made use of a modified version of the non-dimensional acceleration given by equation (2.6), to account for the rotation of both the inner and outer cylinders and also the variations of the fluid viscosity due to shear-thinning. This entailed a nonlinear change in the rotational speed of the cylinders to ensure the same non-dimensional acceleration with respect to their previous work [78]. The experimental protocols are summarized in table 1.

**Table 1.** Summarizing table of the literature on the transitions to EIT.

								transitions	
				method for relaxation time			acceleration protocol	
paper	radius ratio	aspect ratio	polymer		El	β		ramp-up	ramp-down
**boger fluids**		
[24,44,79,80]	0.708	54	PAAM ($M_{w}=5\times 10^{6}g\;{\mathrm{mol}}^{-1}$)	oscillatory rheology/ normal stress decay	0.09–0.16	0.08	variation of El at fixed Re/linear ramps-unknown rate	CF-TVF-RSW-DOs	no hysteresis
					0.16–0.22			CF-TVF-DOs	
					0.22–0.34			CF-DOs	
					0.023–0.033	0.78		CF-RSW-DOs	—
	0.83	73.71			0.025	0.82		CF-TVF-WTVF		
								0.03–0.08				CF-TVF-RSW-DOs	
								0.09–0.15				CF-DOs	
								0.2–0.27			CF-DOs	CF-DW-DOs
[66]	0.883	46.6	PEO ($M_{v}=2\times 10^{6}\;g\;{\mathrm{mol}}^{-1}$)	steady-state rheology	0.002–0.06	0.185–4.32	steady state	CF-TVF-WTVF	no hysteresis
					0.07–0.5	5.32–12.4		CF-RSW-MST	
[69]	0.77	21.56	PAAM ($M_{w}=5.5\times 10^{6}\;g\;{\mathrm{mol}}^{-1}$)	oscillatory rheology	0.2263	0.148	ΔRe/Δτ≤1	CF-TVF-RSW-EIT	—
[23]	0.77	21.56	PAAM ($M_{w}=5.5\times 10^{6}g\;{\mathrm{mol}}^{-1}$)	oscillatory rheology	0.19	0.378	ΔRe/Δτ=0.3312	CF-TVF-RSW-EIT	—
[52]	0.827	30 (free surface)	PIB ($M_{v}=4.2\text{--}5.2\times 10^{6}\;g\;{\mathrm{mol}}^{-1}$)	normal stress decay	0.0562	0.154	steady-state/stepped ramps	CF-TVF-RSW-MST-EIT	CF-RSW-MST-EIT
					15			CF-RSW-FP	—
[47]	0.909	30	PEO ($M_{w}=8\times 10^{6}\;g\;{\mathrm{mol}}^{-1}$)	extensional rheometry (CABER)	0.06–0.09	0.09–0.21	ΔRe/Δτ≤0.6	CF-TVF-RSW-MST-EIT	CF-RSW-MST-EIT
					0.17–0.9	0.48–2.62		CF-RSW-FP-EIT	CF-DW-FP-EIT
[75]	0.8	45.9	PEO ($M=10^{6}\;g\;{\mathrm{mol}}^{-1}$)	Carreau model	0.1	13.49	Steady-state	CF-RSW-FP	—
[78]	0.912	60.7	PEO ($M_{v}=2\times 10^{6}\;g\;{\mathrm{mol}}^{-1}$)	extensional rheometry (CABER)	0.00047	0.3	ΔRe/Δτ≤0.68	CF-TVF-WTVF-MTVF-TTF	—
					0.0017–0.023	0.93–0.78		CF-TVF-WTVF-MTVF-WTVF	—
[15]	0.912	60.7	PEO ($M_{v}=2\times 10^{6}\;g\;{\mathrm{mol}}^{-1}$)	extensional rheometry (CABER)	0.1–0.2	2.82	ΔRe/Δτ≤0.68	CF-TVF-MST-EIT	CF-RSW-MST-EIT
[81]	0.883	46.6	PEO ($M=10^{6}\;g\;{\mathrm{mol}}^{-1}$)	steady-state rheology-CABER	0.16–0.3 (0.079–0.094)	0.57–0.47	ΔRe/Δτ≤0.054	CF-RSW-FP-EIT	—
**shear-thinning fluids**		
[23]	0.77	21.56	XG ($M_{w}=1.76\times 10^{6}\;g\;{\mathrm{mol}}^{-1}$)	oscillatory rheology	1.25	107.81	ΔRe/Δτ≤1	CF-TVF-WTVF	—
					13	50.18		CF-EIT	
					0.14	7.283		CF-TVF/RSW-EIT	
					0.88	6.5		CF-TVF/RSW-EIT-WTVF	
					74	486.86		CF-TVF-SVF-RSW-EIT	
[68]	0.77	21.56	XG ($M_{w}=1.76\times 10^{6}\;g\;{\mathrm{mol}}^{-1}$)	oscillatory rheology	≪1	0.0424	ΔRe/Δτ≤10	CF-TVF-WTVF with merging and splitting	CF-TVF-WTVF hysteresis in the merging and splitting events
					≪1	1.029			
					0.0256	4.354			
					0.0327	6.037			
					0.044	23.46			
					0.883	108.7			
					9.29	743.936			
[70]	0.83	12.97	XG ($M_{w}=1.76\times 10^{6}\;g\;{\mathrm{mol}}^{-1}$)	oscillatory rheology	0.0013	6.38	ΔRe/Δτ≤5.5	CF-TVF-WTVF	—
					0.002	42		
					1	482		CF-TVF	

The spatio-temporal maps can be further processed to produce frequency maps (e.g. [15,23,68,69,78]). This is performed by dividing the spatiotemporal map into segments and by applying fast Fourier transformation. The resulting frequency maps illustrate the dominant temporal or spatial frequencies of the flow, which serve as an additional tool to identify the transitions and characterize the instabilities.

It is worth noting that flow visualization experiments were also conducted by [50–52] by visualizing the mica flakes arrangement in a (r−z) vertical plane across the gap. This was achieved by illuminating the plane with a laser sheet, as would be done for laser-based methods described later, and recording reflected light intensity with a camera, allowing us to qualitatively describe the flow therein.

PIV [82] is an established laser-based flow diagnostic technique that can provide quantitative velocity information. It has been successfully applied in time-resolved or double-frame mode to Newtonian flows with stationary or slowly evolving laminar and moderately chaotic flow states (e.g. [49,83]) as well as fully turbulent flows (e.g. [84]), and particle suspensions [85,86], most of the time in (r−z) vertical planes (see figure 2). The challenge in applying PIV to elasto-inertially turbulent flows resides, as for conventional turbulence, in the high-flow velocities and short-lived flow structures that need to be captured requiring high-speed systems to achieve good temporal resolution [84]. For that purpose, laser Doppler velocimetry (LDV) was used by [53] to access local velocity information within the gap at high temporal resolution. To the best of the authors’ knowledge, PIV has never been applied to characterize the properties of EIT in the TC flow. The qualitative visualizations of [50–52] yet suggest that such measurements could be of great interest.

Closely linked with PIV is PLIF [87], a method typically used to visualize flows or quantify mixing dynamics. In the context of TC flows, it has been mostly applied to Newtonian [42,88,89] and particle-laden Newtonian flows [86] to assess mixing characteristics. Extending such studies to viscoleastic TC flows would be highly beneficial in the future; combined with PIV [43,86,89], they can provide invaluable insights on mixing mechanisms in the presence of viscoelasticity.

Finally, apart from the optical methods, TC flow characterization setups offer the possibility to measure the torque exerted on the rotating cylinder [46–48,85,90,91] (see figure 2). Torque measurements are either used to detect the onset of secondary flows [46,47] or in a more elaborate way to discuss the evolution of friction properties with the variations of control parameters [17,48,84,90]. This can also be used as a way to detect polymer degradation during an experiment [47].

It should be noted that polymer degradation poses a significant experimental challenge and is one of the main reasons that there is limited experimental work on viscoelastic TC flows at high Re (greater than $O(10^{3})$), despite the interesting dynamics that can potentially be uncovered as indicated by numerical studies [39,40,92]. The degradation is more pronounced for flexible and semi-rigid polymers like PAAM and PEO, compared to rigid polymers like XG [74,93]. Other challenges may arise in certain experimental setups as follows: the local viscous heating phenomenon not being fully compensated by external temperature baths; long duration of ramp-up/down experiments needed to ensure the quasi-steady process that may fuel viscous heating and polymer degradation mechanisms, and limitations in TC rotational speed, and hence range of Re achieved (e.g. in rheometer-based test sections).

## 3. Inertia and elasticity in Taylor–Couette flows

In this section, we discuss the transition sequences and flow states encountered in the presence of inertia, elasticity and both.

### (a) Inertia-dominated transitions

In TC flow of inelastic fluids with the inner cylinder rotating only, the relevant control parameter is the Reynolds number (equation 2.1). At very low Reynolds numbers, the flow is purely azimuthal and laminar, and is well known since [1,94] that TVF is the first emerging instability. It is accompanied by an increase in the required torque to maintain the rotational speed of the inner cylinder. TVF is structured by equally spaced vortices along the height of the cell, separated by alternating inflow and outflow boundaries. As the Reynolds number increases, the outflow boundaries become stronger due to centrifugal forces [95]. Secondary and higher-order bifurcations have a non-axisymmetric character: wavy Taylor–vortex flow (WTVF) [13] is a single periodic modulation of TVF, characterized by the appearance of a distinct frequency peak corresponding to the frequency of longitudinal waves. The vortex centres move in the axial direction, in phase with the azimuthally travelling wave but also radially and out of phase with each other [95,96]. Additional frequencies appear on the periodic WTVF when Re increases causing the flow to transition from quasi-periodic [16,97–99], a flow regime termed modulated wavy Taylor–vortex flow (MWTVF) [98,100,101], to chaotic at higher Reynolds numbers. The frequency peaks disappear therein and a broadband component appears in the spectrum instead, denoting the transition to chaotic Taylor–vortex flow (CTVF), wavy turbulent vortex (WTV) and ultimately turbulent Taylor–vortex (TTV) [97,102]. Due to the ambiguous nature of the higher-order instabilities, the transition pathway to turbulence differs in the published literature [77,97]. Several experimental [16,102–106] and numerical [107–111] works address the conventional, inertia-driven turbulent flow in the TC system, summarized in the review of [17]. State-of-the-art TC facilities [112] have been developed and dedicated to the systematic study of turbulent TC flow of Newtonian fluids.

### (b) Purely elastic transitions

In the case of purely elastic transitions, the control parameter is this time Wi (equation 2.4), or alternatively the Deborah number De (see [113]), and it is typically assumed that Re≪1 (or Ta≪1). The first experimental observations of purely elastic instabilities were reported by [114,115]. In the case of vanishing inertia (De=20, $\text{Ta}=9.6\times 10^{-8}$), vortices similar to the Newtonian ones develop, which become unstable after some time, leading to a more complicated structure composed mainly of elastically modified vortices, with smaller axial wavelength than the Newtonian equivalents, and superimposed noisy flows of different spatial scales. By using LDV, the same authors resolved the velocity components of the flow field and noted that the observed viscoelastic instabilities are oscillatory in nature [53].

In a similar study, for a more elastic case (El=2770), [50] reported the abrupt transition of steady and axisymmetric vortices to a chaotic flow for De=21.5, $\text{Ta}=1.11\times 10^{-5}$. During this chaotic flow, vortices illustrate sharp, jet-like boundaries with strong velocity gradients that merge and split. For a less elastic solution (El=44) and also very low inertia (Ta $=1.93\times 10^{-3}$), the abrupt transition from steady vortices leads to axisymmetric migrating bands that originated from the centre of the cell and travelled in opposite directions. This process repeated when these bands reached the top and bottom-end boundaries of the TC cell.

When expanding their work to the case of independently rotating cylinders and both for small and large gap TC geometries [51,52], the same group reported a purely elastic instability of counter-rotating vortices and migrating bands as those instabilities appeared at vanishing Reynolds numbers (Re≪1), for both rotating inner and outer cylinders and for both small and large gaps. However, the elasticity of those fluids was smaller (El =44 and El =15, respectively) than the ones used in their previous work. Steinberg [19] yet noted that discrepancies between the theory of [114] and the experimental results of [50–52,114,115] lead to the conclusion that those experiments were performed in non-isothermal conditions. In these works, the instabilities illustrated large time scales, they appeared at very low Re, and they are composed of axisymmetric vortices that are in contrast to the theory as noted by [116].

Further increasing Wi, the flow becomes increasingly chaotic up to a point where it resembles turbulence with yet negligible inertia and can be called elastic turbulence (ET) [58,117–119]. The work of Groisman and Steinberg [44,67,80,118,120,121] illustrated an alternative path for elastic instabilities. The flow in their experiments was characterized by a hysteretic transition with the abrupt onset of disordered oscillations (DOs) during the ramp-up (increasing Wi) and the appearance of stable, axisymmetric and equally spaced solitons, called solitary vortex pairs or diwhirls (DW) during the ramp-down (decreasing Wi). The hysteresis was also pronounced in torque data, illustrating higher stresses during the ramp-down. DW have been reported to be purely elastic in nature, whereas DOs can be considered purely elastic for large values of El. However, both DW and DO occur in a context of non-negligible Re values. The flow must thus overall be considered elasto-inertial. This is the topic of the next section.

### (c) Elasto-inertial transitions

TC flows in which both inertia and elasticity are non-negligible are now considered. The control parameters become then Re and Wi, or alternatively Re and El. For Boger fluids (viscoelastic fluids with no other apparent complex rheology feature than elasticity), the basic transitions seem to be mainly dependent on fluid elasticity El. In their extensive work, Dutcher and Muller have used high-molecular-weight ($M_{v}=2\times 10^{6}\;g\;{\mathrm{mol}}^{-1}$) PEO solutions in different concentrations and glycerol volume fractions to produce Boger fluids with weakly (0<El<0.023) [78] and moderate (El=0.1−0.2) [15] elasticities. In their first article [78], they reported the transitions in a wide range of Reynolds numbers for both co- and counter-rotating cylinders for weakly elastic fluids. In the case of a stationary outer cylinder and for the less elastic fluid, the transition was Newtonian-like, CF→TVF→WTVF→MWTVF→TTV, as reported in their previous work for Newtonian fluids [77]. The same Newtonian-like behaviour was reported in [66]; they, however, observed an elastically modified WTVF, with an increasing frequency at the onset and decreasing oscillation amplitude for increasing elasticity. For increased elasticity, but still within the regime of weakly elastic fluids, [78] observed a modified Newtonian-like transition sequence, CF→TVF→WTVF→MWTVF→WTVF${\;}_{2}$, in which the WTVF was interrupted by the appearance of a modulated wave for a short range of Reynolds numbers. The two modes of WTVF (WTVF and ${\mathrm{WTVF}}_{2}$) seemed to have the same nature and frequencies. The general effects of low elasticities were reported to be (i) a delay of TVF, as also observed in [122], (ii) a stabilization of the ${\mathrm{WTVF}}_{2}$, extending to a wider range of Reynolds numbers, and (iii) a destabilization of MWTVF appearing in a narrower range of Reynolds number. Most importantly, a delay or total suppression of TTV and modifications in the structural properties of the weakly turbulent regimes were observed. This was accompanied by the appearance of an elastically modified WTVF at high Reynolds numbers Re≅800, which alters the structure of inflows and outflows and suppresses CTVF. This regime is associated with the appearance of a frequency subharmonic, without any other changes in the frequency spectra.

For a stationary outer cylinder and moderately elastic fluids, [15] found that the transition follows the path CF→SV→DRSW→EIT, where SV stands for standing vortices and DRSW for disordered rotating standing waves (RSWs). SV is an axisymmetric state similar to TVF with the same axial wavelength but with a modified structure characterized by broad inflow/outflow boundary regions, whereas DRSW is a disordered state of the grid-like RSW. The transition was found to be hysteretic; for a decreasing rotational speed of the inner cylinder, the SV is suppressed, and the transition follows the path EIT→DRSW→RSW→CF. The increased elasticity in this case also leads to the appearance of EIT. Extending the work to the case of co- and counter-rotating cylinders revealed similar flow patterns [15]. These studies highlight the stark differences between the elasto-inertial and Newtonian TC flow cases, both in terms of critical conditions and flow states encountered, as illustrated in figure 1. In particular, turbulent-like flow, EIT, is found an order of magnitude earlier in terms of Reynolds number than conventional turbulence.

Similar transitions have been reported in several experimental works with Boger fluids of different polymers and polymer viscosity ratios β: PAAM [23,69], PEO [47] and Polyisobutylene (PIB) [52]. However, in those works, no SV was observed during the ramp-up, but rather the CF transitioned to TVF and then to RSW, following the path CF→TVF→RSW→DRSW→EIT. As shown by Groisman and Steinberg [44], for a certain range of elasticities (0.15–0.22), the transition from TVF or SV to DRSW is possible without the appearance of RSW. In some cases, no intermittent TVF is reported [66]. Groisman and Steinberg [44] found that the appearance of TVF can be suppressed for high values of β. Indeed, in their experiments, Crumeyrolle *et al.* [66] have used deionized water as a solvent, resulting in solutions with high values of β and substantial shear-thinning. There is some discrepancy in published works regarding the transitions during ramp-down; [44,66] report a non-hysteretic transition, whereas [47,52] report a hysteretic behaviour with a transition similar to the one by [15]. This can be attributed to differences in polymer solutions and experimental protocol (e.g. acceleration/deceleration of the inner cylinder) employed.

**Figure 4.**
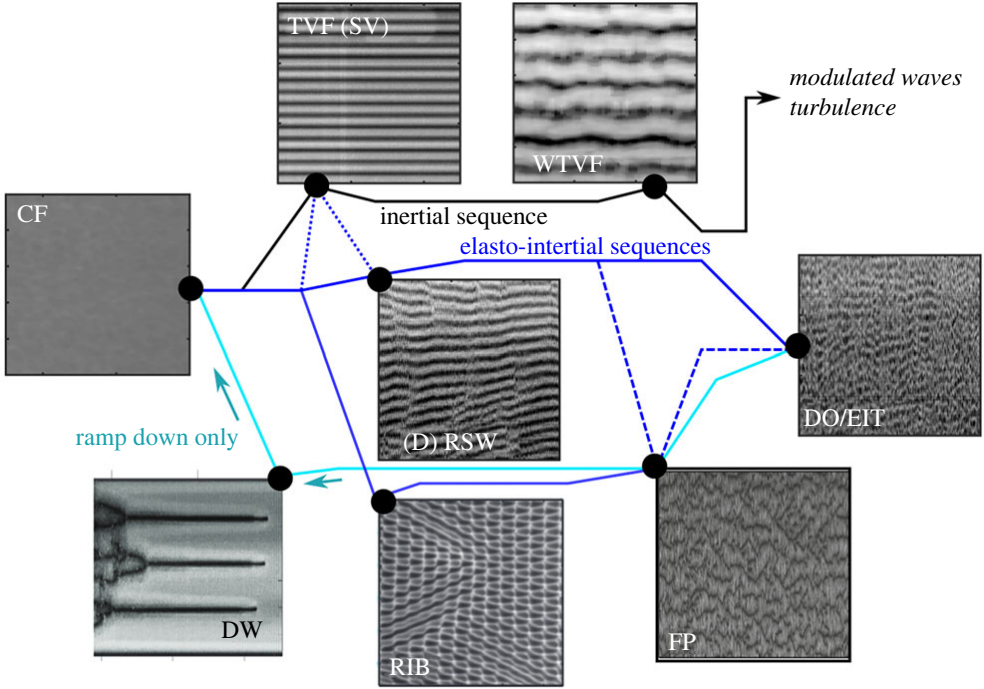
Sketches of various flow states encountered during inertial and elasto-inertial transition sequences. All images are space-time diagrams (with time on the x-axis and the axial distance on the y-axis) constructed from flow visualization, and represent steady-state cases for which the control parameters were constant. Illustrations were taken from [23,45,47,69,81]. (Online version in colour.)

The RSW instability, also called ribbons (RIB), is characterized by two counter propagating axial waves with a distinct frequency peak, found to be associated with elastic waves and elastic frequency respectively, in recent works [69]. The RSW instability is not considered a purely elastic instability [24] but an elastically modified inertial instability. Two different groups have predicted analytically the existence of the RSW instability for Boger fluids, using linear stability analysis of modified Oldroyd-B models, in two different modes: rotating and standing waves (spirals and ribbons, respectively) [55,123,124]. It was later proven by [125] that these structures are also stable above the linear stability threshold. Even though in the literature the two terms (RSW and RIB) are used interchangeably, there is a qualitative difference between the two, as in the RSW case the underlying TVF base structures are more evident compared to the grid-like structure of RIB (see figure 4). The route to EIT through RSW is through merging and splitting events (MST), which lead to a shift in the peak of the elastic frequencies as the role of inertia increases, until the flow becomes fully turbulent when the frequency signature of RSW vanishes [69]. Other transition pathways previously identified were through disordered rotating standing waves (DRSW) [15], defect-mediated turbulence (DMT) [45] and DOs [44]. However, a qualitative difference also exists between these phenomena. MST refers to the merging and splitting induced by the base TC flow (of RSW), whereas DMT refers to dislocations and defects in the structures of the two axial waves (of RIB).

TC flows of higher elasticity Boger fluids exhibit very different properties. The laminar flow transitions to RSW for a very narrow range of Reynolds numbers, but instead of following the path previously described, the waves quickly merge in coherent structures of strong radial inflow jets, called flame patterns (FP), first reported by [51,52] in the case of vanishing inertia. The same group reported purely elastic structures of strong inflows in the earlier work [50] without using the term FP. The transition, in this case, follows the path CF→RSW→FP→EIT (figure 4). FP, comprising structures of solitons, appear chaotic during ramp-up, with their number varying arbitrarily [126]. As the Reynolds number increases, the number of solitons increases through merging and splitting events [51] when the perturbation amplitude reaches a critical value [81]. These merging and splitting events eventually lead to EIT, where their number converges to a constant value [81]. Despite their chaotic nature, it has been reported that the number of FP follows a Gaussian distribution at a given Ta or Re number [81]. It is likely that the FP instability is similar to the DO structures reported in the work of [44,80,120] above the co-dimension point (the critical El, above which Couette flow transitions straight to DOs), as DOs is a very broad term engulfing a spectrum of different phenomena such as MST and EIT. The same authors also reported a transition from CF to DOs via neutral linear oscillations, a structure similar to RSW and spirals.

When a transition via the FP instability path appears, the flow becomes strongly hysteretic [47,67,127] with the appearance of DW, as the rotational speed decreases (ramp-down). This leads to a transition sequence EIT→FP→DW→CF. Recent DNS simulation by [41], however, indicated that the deceleration of the inner cylinder is not a necessary condition to achieve DW, and they can also appear for increasing elasticity at a fixed Re. As their name implies, each DW comprises two concentrated vortices with a narrow but strong inflow boundary between them. Each vortex pair is independent to other solitons, separated from each other by regions of purely laminar Couette flow. This asymmetry between inflow and outflow regions is characteristic of the DW instability [41,67,92]. At the centre of the inflows, the fluid initially accelerates near the outer cylinder, reaches a maximum velocity at the mid-gap, and then decelerates. Although the structures are stable, they can merge if their distance reaches a critical value, which was reported to be five times the gap width by [67]. However, [47] reported that the number, and thus the distance between the DW depends on the deceleration rate of the inner cylinder, suggesting a spectrum of polymer relaxation times $t_{e}$ governing the appearance of DW if the instability is considered purely elastic.

The fact that the velocity profile across the core of the DW is not dependent on the elasticity number and that the DW appear even at very low Re, below the stability limit of the base flow, lead [67] to hypothesize a purely elastic nature for this instability. Thomas *et al.* [125] also observed FP and DW numerically in TCs of purely elastic fluids, enforcing the previous hypothesis.

The appearance of DW is attributed to a phase lag between the velocity gradient $\partial u_{r}/\partial r$ and the polymer elongation when the time scale of the cross-gap movement for a polymer becomes comparable to its relaxation time ($d/u_{r}\approx t_{e}$) [67]. This results in hoop stresses towards the inner cylinder pumping energy into the flow and giving rise to the DW. Another, self-sustaining mechanism for the appearance of DW is proposed by the numerical work of [126,128], suggesting that small perturbations of the azimuthal velocities on the outer cylinder lead to stress gradients, which pull the fluid radially close to the outer cylinder and axially close to the DW core, regenerating the stress gradients. The origin of DW structures was also associated with strong polymer elongation, resulting in high hoop stresses on the outer cylinder by [92,127] in their numerical work, recognizing the outer cylinder as an elastically dominated region of the flow. However, DW structures were predicted to be unstable in both axisymmetric and non-axisymmetric perturbations by [126,128], which is in sharp contrast to the coexistence of coherent structures with highly fluctuating perturbations reported by [92,129].

Since the first experimental observations of FP and DW, surprisingly in the same year [51,67], the two instabilities have been closely linked and sometimes reported to have the same structure [52,127], same inflow/outflow asymmetry [80,92] or merging and splitting mechanism [125]. In addition, both instabilities appear to belong to a separate branch of stationary solutions [126], resulting from hoop stresses on the outer wall [92]. However, FP appears to exhibit continuous power-law decay exponents of radial velocities near the outer wall, similar to those found in EIT, in contrast to those found in the case of DW which illustrate two different exponents at low and high frequencies [92]. This implies different levels of polymer extension in the cases of FP, DW and EIT, which potentially alters the stability of the soliton structures. In the middle of the gap, [92] successfully reproduced the decay exponents found experimentally by [118] for various values of Wi. However, no experimental data on the FP velocity field exist to perform a comparison with the FP/DW/EIT as they are attained numerically. In the recent numerical work of [41], structures resembling FP appeared as a result of a vortex merging and splitting mechanism of elastically destabilized, oscillatory DW, for high values of El. However, they were found to be axisymmetric, in contrast to the experimental observations discussed earlier. According to [129], unsteady DW play a significant role in the structure of ET; the coupling of these vortical structures with the propagation of elastic waves near the inner wall is reported to be the source of the ET energy cycle.

Most of the experimental results presented here examine small-gap TC flows, with radius ratios of $\eta_{\text{cell}}>0.7$ (see table 1). Elastic instabilities depend strongly on the curvature of the streamlines of the base flow, and therefore, the radius ratio can also be expected to have a significant effect in the TC flow, as indicated in numerical works [130]. However, as table 1 indicates, experimental studies in the literature have been performed using a wide range of experimental conditions, making it hard to isolate the effects of $\eta_{\text{cell}}$.

## 4. Modulation of elasto-inertial transitions through fluid complexity

In most relevant industrial and environmental applications, viscoelasticity is not the sole source of nonlinear rheological behaviour and may be combined with several other features: shear thinning, shear banding, yield stress and thixotropy. The fluid may also be more than a simple suspension of polymers in a Newtonian solvent, but also include suspended particles, bubbles or cells. Recent investigations on TC flows have provided insight on how inertial, elastic, elasto-inertial transitions and EIT can be modulated by some of these additional fluid features. This section outlines the effect on elasto-inertial transitions of two such fluid properties: shear thinning and presence of solid particles.

### (a) Shear thinning

Shear thinning, namely, the decreasing viscosity for increasing shear rates, can be described by the β parameter (equation 2.3). However, it is commonly expressed by fitting the shear viscosity data with the Carreau model:

4.1 $$\eta (\dot{\gamma })=\eta_{\infty }+(\eta_{0}-\eta_{\infty }){\left(1+{\left(t_{c}\dot{\gamma }\right)}^{2}\right)}^{(n_{c}-1)/2},$$

where $\dot{\gamma }$ is the shear rate; $\eta_{\infty }$ and $\eta_{0}$ are the infinite and zero shear-rate plateau viscosity, respectively; $t_{c}$ is the Carreau model time scale and $n_{c}$ is the Carreau flow index. A strongly shear-thinning fluid has values of $n_{c}\to 0,$ whereas for Newtonian fluids, $n_{c}\to 1$. However, $n_{c}$ is a free fitting parameter and can lead to inconsistencies. An alternative, more consistent parameter for the estimation of the shear-thinning nature is the mean gradient of the viscosity curve $\bar{n_{e}}$, termed effective flow index ($\bar{n_{e}}=1\to \text{Newtonian}$, $\bar{n_{e}}=0\to \text{purely shear thinning}$) [23,68], obtained from:

4.2 $$n_{e}=\frac{\partial \log(\eta )}{\partial \log(\dot{\gamma })}+1.$$

The presence of shear thinning appears to modify even the simplest case of laminar CF, in which stratification of the viscosity is observed due to the variation of the shear rate across the flow cell. More specifically, a low viscosity is expected near the inner cylinder (high shear rates) and a higher viscosity near the outer wall (lower shear rates) [54,57,131]. The level of base flow stratification is thus highly dependent on the gap width, which may result in different bifurcations between different TC apparatuses [132] and bound to have an effect on higher order elastic or inertial transitions.

Following [23], the shear-thinning mediation of the flow transition to turbulence can be classified into three regimes: (i) highly shear thinning—weakly elastic, a case in which elasticity can be neglected, (ii) non-negligible shear thinning and strong elasticity, and (iii) an intermediate regime between the two.

For the case of vanishing elasticity and substantial shear thinning, the flow transitions follow a modified Newtonian-like path: CF→TVF→WTVF [66,68,70,77]. The previously described viscosity stratified CF gives rise to an also modified TVF, with the vortex cores concentrated towards the outflow boundaries, observed both experimentally [49,54,133] and numerically [131]. However, there is a contradiction in the effect of shear thinning on the strength of outflow/inflow boundaries, with [49] reporting no change, whereas [131] numerically predicts an increase in their relative strength. Cagney and Balabani [49] reported a decrease in the root mean square of the vorticity across the meridional plane for increasing shear thinning. The critical Reynolds number for the transition to the modified TVF seems to decrease for increasing shear thinning for both wide [68,131,134,135] and narrow gaps [49,54]. This general trend contradicts the observations of [135], who reported a critical Reynolds number similar to the Newtonian one for the case of small gaps and those of [132] who reported higher Taylor numbers in the case of non-Newtonian fluids, without however providing details on the rheological properties of the fluids used. A related matter which can lead to inconsistencies or contradictory results regarding the critical Reynolds numbers in the case of shear-thinning fluids is the viscosity used in the definition of the Reynolds number, since the latter varies across the gap [54,57]. Some authors use the nominal shear rate across the gap to calculate the effective viscosity and the Reynolds number [49], whereas others use the zero-shear viscosity [57], the viscosity at the inner cylinder (maximum shear-rate) or an averaged cross-gap viscosity based on the velocity profile [54,136].

The number of vortices has been found to decrease (increased wavelength) as a result of shear-thinning, as reported experimentally by [49,70] for both wide and narrow gaps. This contrasts the numerical works of [136,137] which predicted an increase in the wavelength in the case of narrow gaps and a decrease in the case of wide gaps. The critical Reynolds number for the onset of WTVF was found to vary non-monotonically with the extent of shear-thinning index [49,70] and delayed for increasing polymer concentration [68]. A change in the structure of the WTVF instability with polymer concentration and shear-thinning was also reported. Unlike the Newtonian case, in which the flow oscillations are concentrated at the inflows at mid TC height, in the viscoelastic one, both inflows and outflows oscillate with comparable amplitudes, which is higher compared to the Newtonian counterpart. However, as both elasticity and shear-thinning increase by increasing polymer concentration, this effect cannot be attributed to one of the two properties.

**Figure 5.**
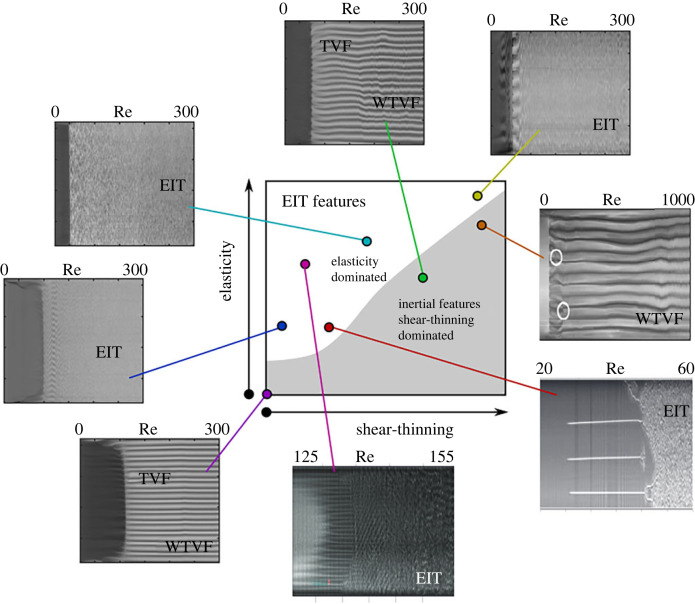
Illustration of the modulation of EIT by shear thinning, inspired and adapted from [23,47,68,138]. (Online version in colour.)

For fluids with substantial shear-thinning and elasticity, the transition can surprisingly remain Newtonian-like (see figure 5, [49,68,70]), but the onset of TVF is delayed [70]. However, the stability of TVF weakens as the elastic effects become more important [57], which leads to a drift of the base flow along the axis of the cylinder [54,133]. The drift causes variations in the wavelength as the Reynolds number increases and is closely linked to merging and splitting events of TVF [57,68]. According to [68], there are two different modes in the merging and splitting of vortices depending on whether the flow is steady or unsteady (before or after the onset of WTVF). In the first case, the merging happens via a gradual decrease in the size of the vortices, whereas in the second case, the mechanism involves a combination of waviness and vortex drifting, associated with a more chaotic nature and the introduction of instabilities in the WTVF. Moreover, this mechanism has been found to be strongly hysteretic as it could be observed even at low Reynolds numbers, close to the critical value for the onset of TVF, during ramp-down. However, the unsteady mode of vortex merging and splitting events could not be observed during ramp-down. Topayev *et al.* [57] have been able to capture both experimentally and numerically the merging and splitting of steady TVF, attributed to the presence of axial perturbations in the flow, and suggested a possible mechanism based on a generalized Eckhaus instability, which results in merging or splitting events when the axial wavelength is too small or too large, respectively.

Between these two extremes of shear-thinning and viscoelasticity lies a regime with competing effects which results in an unusual combination of many of the previously discussed instabilities. Experimentally, it is thus necessary to be able to tune independently both the elasticity and the shear thinning, which can be achieved by varying the polymer type, molecular weight as well as the solvent. In their systematic work, [23] investigated the effects of these two parameters and compared them with Newtonian and Boger fluid cases (see figure 5). For a highly elastic, moderately shear-thinning fluid (El =13, $n_{e}=0.71$), the flow is abruptly transitioned from CF to EIT, possibly in a similar path through FP as described for highly elastic Boger fluids. The only other known case of highly shear-thinning fluid transitioning straight to EIT is reported by [75], through FP. For the case of moderate elasticity and shear thinning, the transitions varied from a combination of Newtonian transitions with mixed RSW/WTVF and two modes of WTVF (one inertial and one elastically modified) for the case of (El =0.14, $n_{e}=0.73$) to a Newtonian transition interrupted by EIT in a small range of Reynolds number (El =0.88, $n_{e}=0.85$). Very different phenomena were observed in the case of highly elastic and high shear-thinning properties (El 4=74, $n_{e}=0.56$), in which the flow transitions follow the sequence CF→TVF→SVF →RSW→EIT, where SVF stands for spiral vortex flow. SVF was firstly observed in counter-rotating Newtonian flows [14,139] but has also been predicted analytically as an elastic bifurcation along with RSW/RIB. The previous observations of SVF by [44,45] did not illustrate a clear SVF structure; instead, the SVF appeared as partially overlapping waves in the RSW grid, linked to the DOs or the DMT. Only recently, [54] observed SVF on the upper part of the TC cell in their shear-thinning fluid experiments, superimposed to TVF and leading to RSW. In their case, the transition followed the path CF→SVF/TVF→RSW/TVF→MWTVF.

The most apparent effect of shear thinning seems to be the global delay or suppression of elastic effects [23,68]. The simplest explanation for this observation is the local decrease in viscosity and elasticity number in areas of high shear rate. However, as noted by [23], the effect of shear thinning on the base CF is expected to promote elastic instabilities, if a decrease in the effective gap width is considered due to the high shear rate close to the inner cylinder and the resulting radial viscosity gradient. The same authors reported that for higher-order instabilities (e.g. TVF), which can lead to the propagation of axially moving, elastic waves, the mechanism of shear thinning is more complicated and can be associated with establishing preferential flow paths, observed also in the suppression of elastic instabilities in other geometries [140]. These flow paths can lead to the damping of the axial elastic waves and radial paths becoming predominant. In that sense, shear thinning adds nonlinearities in the interplay between elastic and inertial stresses. However, as suggested by [141], this interplay is not sufficient to account for the effect of shear thinning as even for the case of shear thinning without elasticity, nonlinearities remain due to the shear rate dependence of the viscosity.

Finally, it is worth mentioning that recent interest in the non-Newtonian fluid mechanics community has been found in Wormilke Micellar Solutions, which have been used in TC devices as elastic, sometimes shear-thinning [61,138], but mostly shear-banding [58,117] fluids. The interplay between elastic instability and shear banding then arises at the interface between the flowing inner region and non-flowing outer region. A combination of bulk and interfacial disturbance mechanisms is then found to alter the onset of ET at low Re [58,117].

### (b) Particle suspensions

**Figure 6.**
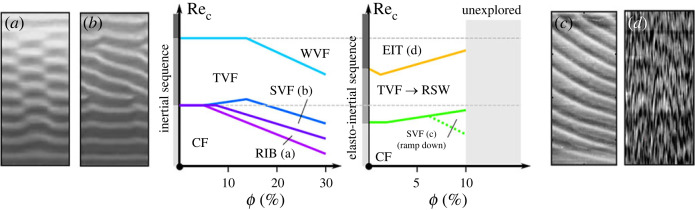
Simplified representation of the inertial (a,b) and elasto-inertial (c,d) transition sequences in particle loaded fluids. Images shown in a,b are inspired from [48,85,143] among others and show that particles destabilize first- and second-order flow states. RIB and SVF states are illustrated by images extracted from [143] and rescaled (*a*,*b*). Images shown in (*c*,*d*) are inspired from [146] and shows that, on a smaller particle volume fraction range explored, particles on the other hand tend to stabilize elasto-inertial flow states. (Online version in colour.)

Recent experimental efforts have been made in trying to understand the role of suspending solid particles on transitions in TC flow, mostly focusing on (i) suspensions in Newtonian solvents and/or (ii) the one-way coupling of the effects of flow on the particle motion. Neutrally buoyant spherical particles have been shown [48,85,90,142–145] to have various effects on inertial transitions in Newtonian TC flows, from primary instabilities up to turbulent flows, among which destabilizing flow states and promoting non-asymmetric modes (see figure 6a,b) seem to be the most prominent. Particulate flows are generally known to exhibit particle migration phenomena. The latter are of significant interest in the fluid mechanics and rheology communities and hence extensively studied [147–149], but are beyond the scope of the present review. Studies of particle migration in TC, arising from inertial [142,144,150] or elastic effects [147], have been so far mostly limited to low particle volume fractions of neutrally buoyant spherical particles in laminar and stable flow conditions.

The effect of particle loading on elasto-inertial transitions and EIT remains to be explored and has only been addressed recently in [146], where it was found that the presence of particles, even at low volume fractions, modulates transition to EIT. This was manifested through (i) an increase in the critical Reynolds number for transition to EIT, (ii) an increasingly hysteretic behaviour with additional flow states, and (iii) an altering of the spectral properties of EIT (see figure 6c,d).

## 5. Summary and perspectives

**Figure 7.**
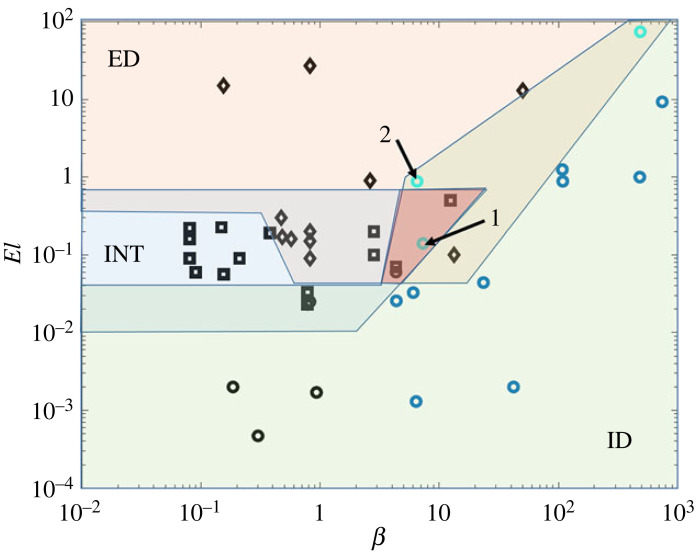
Elasticity number—viscosity ratios as extracted from the literature. Circles denote Newtonian transitions characterized by variations of the CF→TVF→WTVF pathway, squares denote moderately elastic transitions for Boger fluids incorporating CF→TVF→RSW→MST→EIT as a basic path and diamonds correspond to highly elastic transitions with FP, following CF→RSW→FP→EIT. Black markers correspond to Boger fluids, blue markers to shear-thinning fluids and cyan markers to highly elastic-highly shear-thinning cases. ED is the elasticity-dominated regime, ID is the inertia-dominated regime and INT is the intermediate regime. (Online version in colour.)

The published experimental works on TC transitions to EIT are summarized in table 1. To facilitate the disentanglement of the different effects discussed earlier, the results are also plotted in figure 7 in the El−β parameter space. This allows us to illustrate the effect of elasticity for both Boger and shear-thinning fluids, as Boger fluids usually have low values of β (dilute solutions), whereas shear-thinning ones have higher values depending on how strong the shear-thinning effect is. The parameter β was calculated for the shear-thinning fluids using the mean viscosity. Three distinct areas can be observed in the graph:

— The green area is inertia-dominated (ID) characterized by Newtonian-like transitions at low values of El and increasing modifications of the basic transition for increasing elasticities.
— The red area is elasticity dominated (ED) which includes experimental results reporting purely elastic instabilities (FP, DW).
— The blue area is the intermediate region (INT) in which the elastic and inertial effects compete, resulting in a transition with the appearance of RSW. Surprisingly this regime includes only moderately elastic, non or slightly shear-thinning Boger fluids.

The plot clearly illustrates the suppressing effect of shear thinning on the elastic instabilities as reported by [23,68] as at high values of β, the ID region extends to high values of El. In the boundary between ED and ID, the experimental data of [23], denoted by cyan circles, exhibit a strong variety of co-existing elastic and inertial instabilities. The boundaries between ED and INT on the other hand are not very clear, as interpenetrating areas exist: the FP also appears at moderate elasticities (El<1) and close to the semi-dilute regime (β>1). This fact may suggest that the weak polymer entanglement without substantial shear thinning, normally obtained for PAAM solutions, can enhance the elastic behaviour of the fluids. Another explanation is due to the different methods and inconsistencies in the definition of polymer relaxation time and El. The graph also implies the existence of a triple co-dimension point (TP) in the chart at which there is a balance among inertial, elastic and shear-thinning effects. At this point, TVF, RSW and FP could have a significant role in the same transition sequence.

Point ‘1’ in figure 7 illustrates a TVF/RSW coexistence without the appearance of FP in the transition. This implies that this point is more of a co-dimension point between shear-thinning and moderately elastic fluids. Instead, point ‘2’ in figure 7 illustrates all TVF, RSW, and EIT and is closer to the TP point discussed earlier. The fact that point ‘2’ (TP) lies outside the overlapping region of ID, ED and INT implies a distorted shape of the graph due to experimental inaccuracies and inconsistencies.

An open matter is still the nature of EIT as it consists of both inertial and elastic effects. The indications so far point toward a strong similarity between EIT and ET [118,151]. The nature and mechanism of ET and EIT are reviewed by [19], covering the whole spectrum of published experimental and numerical works to date. In their recent article, [129] implemented the first three-dimensional DNS study to highlight the mechanistic differences between EIT and ET. To this end, the authors argue that EIT arises as a result of the interaction between polymer shear stresses and shear flow, with the first one being the dominant contributor to the total turbulent kinetic energy. On the other hand, ET arises from the chaotic events of the polymer extension and relaxation caused by the interaction of unsteady DW and fluid perturbations.

However, to the best of our knowledge, quantitative characterization of the velocity fields in ET and EIT TC flows has only been achieved numerically to date. In-plane flow visualization by [50–52] allowed for an early qualitative experimental description of what were later described (numerically) as near-wall Görtler vortices [39,130], albeit for a different Re range. This highlights the need for more thorough experimental PIV-based investigations to complement recent DNS simulations [39–41,92,152] on the role of elasticity and its interaction with inertia-driven effects. A significant part of the effect of non-negligible inertia on the nature of ET is the modification of the TDR regime through measurements of friction and torque characteristics, expanding the work of [48] on non-colloidal particle suspensions. Elucidating ET/EIT characteristics is not only of fundamental importance but can have significant implications for numerous applications involving polymer processing; it can be harnessed to enhance the manufacturing of advanced materials and to engineer complex fluid rheologies to control and modulate processing flows.

## Data Availability

This article has no additional data.
